# Synthesis and characterization of bimetallic nanocomplex: Application for electrochemical determination of idarubicin as anti-cancer drug

**DOI:** 10.5599/admet.3342

**Published:** 2026-06-02

**Authors:** Thekrayat Joodi Jassim, Raheem Kubaish Barid, Haider Dakhal Hamza

**Affiliations:** 1 College of Education, Department of Chemistry, University of Sumer, Thi_Qar, Iraq; 2 Al-Rifai High School for Outstanding Students, Al-Rifai Education Department, Dhi Qar Education Directorate, Iraq; 3 Al-Qadisiyah Education Directorate, Iraq

**Keywords:** Scanning electron microscopy, solvothermal process, antibacterial behaviour, screen-printed graphite electrode

## Abstract

**Background and purpose:**

Cancer chemotherapy with antineoplastic drugs destroys cancer cells through complete cell death or stops cell development while protecting healthy cells from damage. Monitoring anticancer therapeutics in blood and tissues is important for determining their fate.

**Experimental approach:**

A novel binuclear complex with the formula [Cu(opd)_2_(H_2_O)(μ-SCN)Ni(opd)(SCN)_3_] (opd = o-phenylenediamine) was synthesized and characterized using Fourier transform infrared spectroscopy and UV-Vis spectroscopy. The solvothermal method was used to create a nanoscale version of the binuclear complex. X-ray powder diffraction, scanning electron microscopy, UV-Vis and FT-IR spectroscopy were used to characterize the nanocomplex. The solvothermal approach produced a nanocomplex with an average size of around 56 nm. The complex was tested for its antimicrobial properties on Gram-positive *Staphylococcus aureus* and *Enterococcus faecalis*, as well as Gram-negative *Escherichia coli* and *Pseudomonas aeruginosa*. Additionally, a screen-printed graphite electrode modified by a synthetic nanocomplex (Cu-Ni/SPGE) was presented as an improved electrochemical sensor for the detection of idarubicin (IRN).

**Key results:**

This sensor has a linear dynamic range from 0.01 to 3.0 μM and an impressive limit of detection of 0.003 μM under ideal testing circumstances.

**Conclusion:**

The researchers used a Cu-Ni/SPGE system to perform electrochemical measurements on IRN. The Cu-Ni/SPGE outperformed the untreated SPGE in terms of IRN oxidation. The antibacterial activity of the complex was also studied.

## Introduction

Cancer chemotherapy with antineoplastic drugs destroys cancer cells through complete cell death or stops cell development while protecting healthy cells from damage [1]. Antineoplastic drugs currently aim to exploit this cancer cell distinction for targeted therapy, but most drugs cause adverse effects by attacking both cancer cells and healthy tissues, including bone marrow and hair follicle cells [1]. All cytotoxic drugs used in cancer treatment affect deoxyribonucleic acid (DNA) synthesis [2].

Researchers have developed idarubicin (IRN) as a cytotoxic antibiotic for treating cancers like leukaemia, myeloma and haematological diseases [3]. IRN is a synthetic compound that demonstrates greater lipophilicity than doxorubicin while sharing an anthracycline structure and its mechanism of action includes intercalation among DNA base pairs and inhibiting topoisomerase II [4]. The development of a quick, inexpensive, practical sensor system to measure IRN levels in pharmaceutical products and human biological samples is a high-priority need. Electroanalytical methods stand out as the most effective and widely used techniques for measuring compounds because they deliver high sensitivity and precision, dependable results, and low operating costs [5-8].

Screen-printed electrodes (SPEs) are essential components for electrochemical method development because their manufacturing process enables the production of disposable electrodes with three electrodes printed in a compact design, thereby enabling system miniaturization, real-time analysis, and multiple simultaneous tests under actual field conditions [9]. The production of SPEs requires different conductive inks to be printed onto plastic, textile, and ceramic substrates [10]. The technology achieves widespread use in electrochemical monitoring because it serves environmental and biomedical and industrial monitoring requirements. Commercially available SPEs come in multiple types, but they can also be produced via screen printing, as the method is affordable and easy to use [11]. The research uses modified carbon SPEs to analyse drug concentrations in various samples [11].

The electrode serves as a catalyst, facilitating the flow of electric charge during electrochemical processes [12,13]. Transition metal-metal-oxide sensors exhibit excellent performance, detecting analytes with high sensitivity and selectivity while responding quickly to changes in glucose levels, owing to the oxide's surface layer, which enables multiple-electron oxidation [14]. Copper is available in large quantities and Cu oxides provide more stable properties than their metallic forms [15].

Nickel-based nanoparticles, which display excellent biological compatibility, possess extensive surface area, maintain stable properties, exhibit high electrical conductivity, and demonstrate electrocatalytic capabilities, have been used to enhance both sensitivity and detection limits in electrochemical research [16].

The study presents an easy-to-use electrochemical sensor system that detects IRN with higher accuracy by using a mixed Cu-Ni complex [Cu(opd)_2_(H_2_O)(μ-SCN)Ni(opd)(SCN)_3_] (opd = o-phenylenediamine) on a screen-printed graphite electrode (SPGE). The Cu-Ni/SPGE sensing platform outperformed unmodified SPGE in electrochemical performance for IRN detection. The proposed sensor demonstrated excellent electrochemical sensing performance for IRN detection, providing scientists with accurate measurements across its entire detection range. The antibacterial activity of the synthesized nano-complex (a) was tested on two groups of *Staphylococcus aureus* (*S. aureus* ATCC 25923) and *Enterococcus faecalis* (*Enter_faeca* ATCC 29212) and two bacterial types of *Escherichia coli* (*E. coli* ATCC 25922) and *Pseudomonas aeruginosa* (*P. aeruginosa* ATCC 27853). The results showed that among the synthesized nanocomplexes, showed higher antimicrobial activity than the ligand.

## Experimental section

### Materials and physical experiments

All solvents were purchased from Merck. There was no need for further purification because all chemicals purchased for the experiment were of reagent-grade purity. Sigma-Aldrich provided ortho-phenylenediamine, sodium thiocyanate, copper(II) chloride, and nickel(II) nitrate. Using a Perkin-Elmer FT-IR spectrophotometer model spectrum two, FT-IR spectra were measured from 4000 to 400 cm^−1^ using KBr discs. To analyse absorption spectra, the Optizen-view 2120 UV plus spectrometer was run at room temperature.

The minimum inhibitory concentration (MIC) of an antimicrobial agent is determined using a broth microdilution assay. Serial twofold dilutions of the test compound are prepared in a suitable growth medium in microtiter plates and inoculated (Shimadzu) with a standardized suspension of gentamicin and amikacin. The plates are incubated at 35 to 37 °C for 24 h and the concentrations were determined by the spectrophotometer Optizen 2120 UV plus, made in Korea. The MIC is defined as the lowest concentration of the compound required to prevent visible microbial growth relative to the growth control.

Electrochemical tests were conducted to assess the effectiveness of the proposed sensor in detecting IRN using an Autolab/PGSTAT302N potentiostat/galvanostat from Metrohm, The Netherlands. For electrochemical testing, the commercial SPGEs (DS-110, DropSens, DRP-110, Asturias, Spain) were employed to construct three electrodes on a single planar ceramic substrate: a carbon working electrode (WE), an Ag pseudo-reference electrode (RE) and a carbon counter electrode (CE).

### Synthesis of [Cu(opd)_2_(H_2_O)(μ-SCN)Ni(opd)(SCN)_3_]

**Figure 1.**
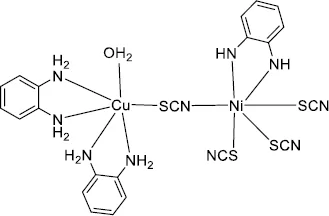
Structures of [Cu(opd)_2_(H_2_O) (μ-SCN)Ni(opd)(SCN)_3_]

In separate beakers, 0.09 g (0.50 mmol) CuCl_2_·2H_2_O and 0.11 g (1.0 mmol) orthophenylenediamine (opd) were dissolved in minimal methanol to form the cationic portion. The opd ligand solution was then added dropwise to a round-bottom flask containing the copper(II) chloride solution, which was stirred and refluxed at 60 °C for five hours. In separate beakers, 0.15 g (0.50 mmol) of nickel(II) nitrate, 0.16 g (2.0 mmol) of sodium thiocyanate salt and 0.05 g (0.50 mmol) of opd were dissolved in a minimal methanol solution to make the anionic portion. The round-bottom flask was then filled with the nickel(II) nitrate solution. The opd ligand solution and sodium thiocyanate solution was then added dropwise while stirring, and the flask was refluxed for five hours at 60 °C. Afterward, the cationic and anionic solutions were mixed and refluxed for 5 h. The beaker's contents were then filtered and allowed to dry entirely at room temperature. The complex structure is shown in Figure 1.

### SPGE modification using Cu-Ni nanocomplex

To make a homogenous solution for the SPGE modification procedure, 1.0 mg of the Cu-Ni nanocomplex was first dissolved in 1.0 mL of deionized water (1 mg mL^-1^ suspension). Subsequently, 3.0 μL of the Cu-Ni nanocomplex aqueous solution was applied to the WE in the SPGE, and the solvent was progressively removed under ambient conditions. They were considered Cu-Ni/SPGEs.

## Results and discussion

### Characterization of complex

**Figure 2.**
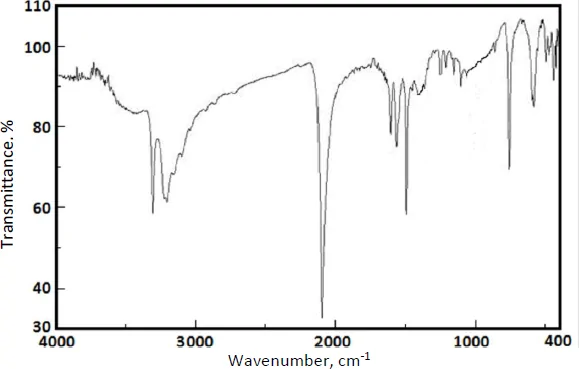
FT-IR spectrum of [Cu(opd)_2_(H_2_O)(μ-SCN)Ni(opd)(SCN)_3_)]

Figure 2 displays the FT-IR spectra of [Cu(opd)_2_(H_2_O)(μ-SCN)Ni(opd)(SCN)_3_]. The amine (NH_2_) group in the free ligand o-phenylenediamine was found to stretch at a frequency of around 1449 cm^-1^ [17]. This spectrum was detected in the combination with [Cu(opd)_2_(H_2_O)(μ-SCN)Ni(opd)(SCN)_3_] at around 1339 cm^-1^. This finding suggests that the stretching frequency of the NH_2_ functional group has decreased from that of the free ligand to that of the complex, which may be due to coordination of the NH_2_ group to copper and nickel ions. Because nitrogen and metal share the non-bonding pair of electrons of the NH_2_ group during the coordination process, the electron density on nitrogen lowers, which in turn decreases the electron density in the N–H bond.

The C=C stretching frequency of the aromatic ring in free o-phenylenediamine was observed between 1640 and 1680 cm^-1^, matching the complex's absorption at approximately 1660 cm^-1^. The stretching frequency of the aromatic C-H groups in free ligand o-phenylenediamine was about o-phenylenediamine in the complex [Cu(opd)_2_(H_2_O)(μ-SCN)Ni(opd)(SCN)_3_]. The 1050 absorption band is due to C-N bond stretching vibrations because nitrogen is bonded to the metal through its lone pair of electrons. A strong absorption peak at 1760 cm^-1^ exists as the C=S bond of the thiocyanate ligand stretches while the thiocyanate ligand connects to nickel metal through its nitrogen atom as a terminal ligand [18].

**Fig. 3.**
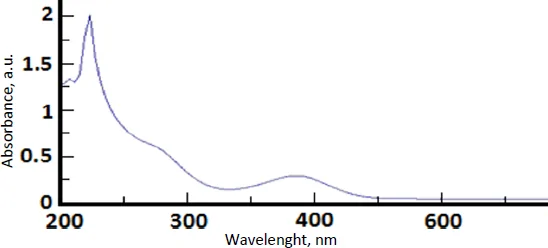
UV-Vis spectra of [Cu(opd)_2_(H_2_O)(μ-SCN)Ni(opd)(SCN)_3_)]

UV-Vis spectra of the complex [Cu(opd)_2_(H_2_O)(μ-SCN)Ni(opd)(SCN)_3_], which appeared in Figure 3, were recorded at two different concentrations because of the existence of two electronic transitions: ultraviolet transitions at 10^-5^ M and visible transitions at 10^-3^ M, both in dimethylformamide solvent [19]. The electronic spectrum of this complex shows two absorption bands at 380 nm and 445 nm due to d-d transitions of the nickel(II) ion [19].

The absorption bands at 210, 225 and 290 nm are related to intraligand transitions n→π*, π→π*, which occur in the orthophenylene diamine ligand and thiocyanate ligand. The electronic spectrum of this complex shows a single absorption band at 425 nm, which corresponds to d-d transitions of copper(II) ion [20].

### Synthesis of nano-complex

25 milliliters of DMF solution were used to dissolve 0.50 mM of [Cu(opd)_2_(H_2_O)(μ-SCN)Ni(opd)(SCN)_3_]. After that, it was ultrasonicated for around half an hour. Following that, the compound's solvothermal reaction was conducted in an oven-mounted autoclave at 120 °C for 48 hours. The precipitates were collected after the solvothermal reaction was complete and the autoclave had cooled to room temperature. Lastly, the [Cu(opd)_2_ (H_2_O)(μ-SCN)Ni(opd)(SCN)_3_] were made by repeatedly washing the precipitates with DMF and ethanol and drying them overnight at 100 °C. After filtration, the precipitate was allowed to dry open. mp: 245 °C; yield: 87 %.

### Characterization of nano complex of [Cu(opd)_2_(H_2_O)(μ-SCN)Ni(opd)(SCN)_3_)]

**Figure 4.**
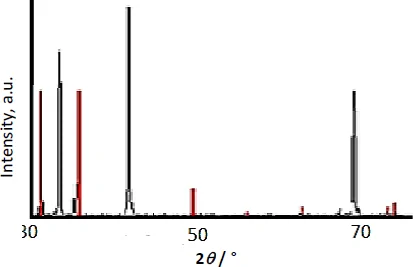
XRD pattern of the nano-complex [Cu(opd)_2_(H_2_O)(μ-SCN)Ni(opd)(SCN)_3_)]

Figure 4 shows the XRD pattern of the nano-sized compound, which was acquired using single-crystal X-ray diffraction. The complex's composition and crystalline phase are revealed by the XRD pattern. The diffraction peak widths indicate that the particles in the nanocrystal complex are nanoscale. The Debye-Scherrer equation of the nanocrystal complex (a) yielded an average diameter of around 56 nm.

**Figure 5.**
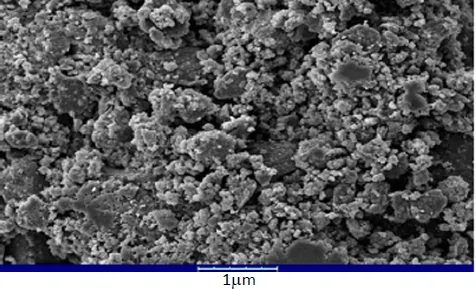
FE-SEM images of the nano-complex [Cu(opd)_2_(H_2_O)(μ-SCN)Ni(opd)(SCN)_3_)]

Figure 5 displays the FE-SEM image of the [Cu(opd)_2_(H_2_O)(μ-SCN)Ni(opd)(SCN)_3_]. The SEM image show that the nano-complex crystals are produced in spheres with smooth surfaces.

### Electrochemical response of Cu-Ni/SPGE compared to unmodified SPGE for IRN determination

Cyclic voltammograms of 1.0 μM IRN recorded at the unmodified SPGE and the Cu-Ni/SPGE in phosphate buffer solutions are shown. The cyclic voltammogram, which showed an oxidation peak at 790 mV with an *I*_pa_ of 2.7 μA, indicated moderate reactivity of the unmodified SPGE toward IRN oxidation. Nonetheless, a clear IRN oxidation peak was visible in the Cu-Ni/SPGE. Apart from the notable rise in IRN's *I*_p_ = 6.9 μA, the SPGE's surface was modified to lower the anodic peak potential (*E*_p_ = 690 mV).

**Figure 6.**
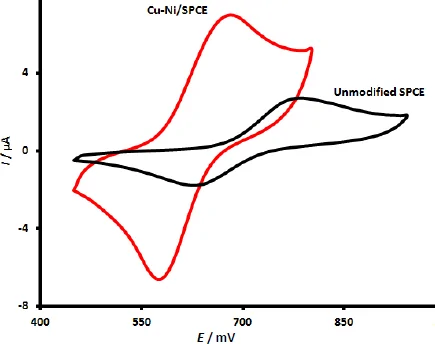
The CV responses of 1.0 μM IRN/PBS at unmodified SPGE and Cu-Ni/SPGE are shown. Scan rate 50 mV s^-1^

### Impact of scan rate

**Figure 7.**
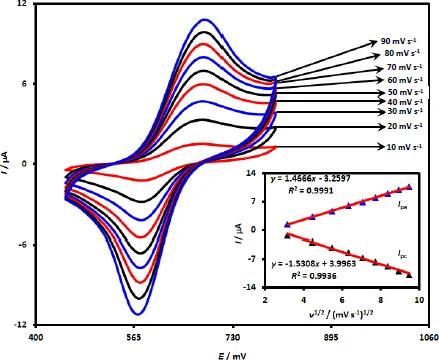
The CV responses of Cu-Ni/SPGE at different scan rates in the buffer solution containing 1.0 μM IRN. Inset: *I*_p_ and *v*^1/2^ have a linear relationship

The Cu-Ni/SPGE response to the oxidation of 1.0 μM IRN in buffer solution was recorded at various scan rates (Figure 7). The CVs shown in Figure 7 indicate that the current height increases gradually with increasing scan rate. The proper linearity between *I*_pa_ and *v*^1/2^ in Figure 7 (Inset) indicates diffusion-controlled electrooxidation of IRN on Cu-Ni/SPGE.

### Chronoamperometric investigations of IRN at Cu-Ni/SPGE

**Figure 8.**
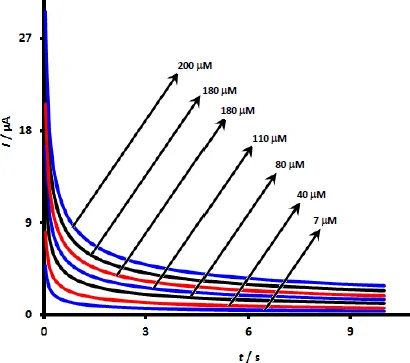
chromatograms for the oxidation of IRN concentrations on the Cu-Ni/SPGE

The oxidation of IRN on the Cu-Ni/SPGE was further investigated using chronoamperometry. The chronoamperograms shown in Figure 8 were recorded on the Cu-Ni/SPGE for different IRN concentrations in the buffer solution using a step potential to 740 mV. The acquired chronoamperograms showed that increases in anodic current were associated with higher IRN concentrations. In chronoamperometric studies, the diffusion coefficient (*D*) of electroactive compounds can be computed using the Cottrell Equation (1):



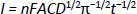

(1)


**Figure 9.**
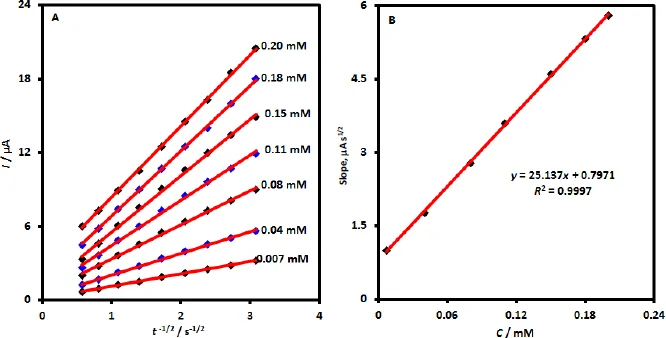
(A) Chronoamperogram plots of *I vs. t*^−1/2^; (B) a plot of the straight-line slope against IRN concentration

The Cottrell plots (*I*-*t*^−1/2^ curves) displayed a linear relationship over a certain time period (0.1 to 3.0 s) (Figure 9A). The Cottrell curves were generated from chronoamperograms recorded at a specific IRN concentration. Next, the slope of the Cottrell curves was plotted against the different concentrations of IRN (Figure 9B). Finally, using Cottrell's equation and the slope of the resulting figure in Figure 9B, the *D* for IRN was found to be 5.4×10^−5^ cm^2^ s^-1^.

### Electroanalysis performance of Cu-Ni/SPGE for *IRN*

**Figure 10.**
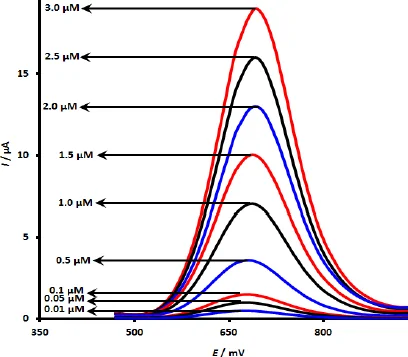
*Cu-Ni/SPGE* responses to different IRN concentrations in the buffer solution

**Figure 11.**
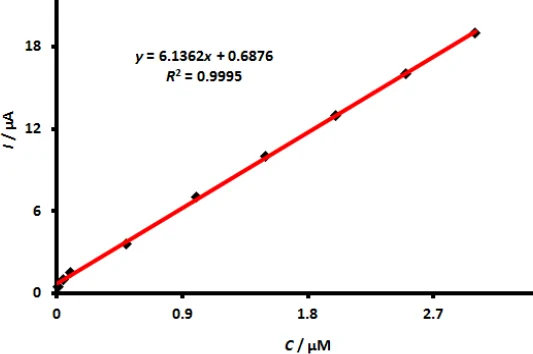
Calibration plot of IRN at Cu-Ni/SPGE

The differential pulse voltammetry (DPV) was used to quantitatively assess IRN on the Cu-Ni/SPGE. The DPVs of Cu-Ni/SPGE at different IRN concentrations in the buffer solution are displayed in Figure 10. As the IRN concentration increased, a linear relationship between the *I*_pa_ and IRN concentration was seen (linear range: 0.01 to 3.0 μM) (Figure 11). Furthermore, the calculated LOD of the Cu-Ni/SPGE for IRN was 0.003 μM.

### Stability, reproducibility, and repeatability studies of Cu-Ni/SPGE

The Cu-Ni/SPGE testing process evaluated its stability over a 15-day storage period at room temperature. The sensor measured its current response to 1.0 μM IRN in a buffer solution every 3 days, reaching the 15-day testing period. The peak current after 15 days decreased by 4.1 % from the sensor's original measurement. The DPV technique was used to assess repeatability by measuring the response current of five modified SPGEs in a buffer solution containing 1.0 μM IRN. The electrodes produced current responses, resulting in a relative standard deviation (RSD) of less than 3.3 %. Ten consecutive voltammetric (DPV) tests with 1.0 μM IRN were performed to evaluate the repeatability of the Cu-Ni/SPGE response. After ten measurements, 96.1 % of the initial response current was retained. The results proved that the Cu-Ni/SPGE sensor demonstrated excellent repeatability and maintained its performance across different testing conditions.

### Real sample analysis

Standard addition methods were used to assess IRN levels in injection samples and evaluate the applicability of the Cu-Ni/SPGE sensor to real samples. The results are presented in Table 1. Recovery of IRN increases from 97.1 to 104.5 %. The study found that IRN detection in samples could be achieved through the developed sensing platform.

**Table 1.** Application of Cu-Ni/SPGE in determination of IRN in real sample (*n*=5)

Spiked concentration, μM	Found Concentration, μM	Recovery, %	RSD, %
0	0.19	-	3.1
0.5	0.67	97.1	2.4
0.7	0.93	104.5	1.5
0.9	1.08	99.1	2.3
1.1	1.31	101.5	2.7

### In-vitro antimicrobial experiment

The antibacterial activity of the complex was tested against two Gram-positive bacteria, *Staphylococcus aureus* (*S. aureus* ATCC 25923) and *Enterococcus faecalis* (*Enter_faeca* ATCC 29212), and two Gram-negative bacteria, *Escherichia coli* (*E. coli* ATCC 25922) and *Pseudomonas aeruginosa* (*P. aeruginosa* ATCC 27853). The broth macro-dilution method was used to determine both minimum bactericidal concentration and minimum inhibitory concentration of the nano-complex (a) (Table 2). The measurement process included determining the size of every bacterial-growth-free inhibition zone (IZ) (Table 2). Three separate MIC, MBC, and inhibitory zone tests were performed before averaging the results. The testing process used amikacin and gentamycin as standard antibacterial agents, which had been tested to establish their effectiveness [21].

**Table 2.** The data of MIC and MBC of opd and the nano-complex [Cu(opd)_2_(H_2_O) (μ-SCN)Ni(opd)(SCN)_3_] against investigated bacterial strains

	opd		Nano-complex	
	MIC, mg mL^-1^	MBC, mg mL^-1^	MIC, mg mL^-1^	MBC, mg mL^-1^
*Escherichia coli*	0.0122	0.0247	0.0121	0.0248
*Pseudomonas aeruginosa*	0.0244	0.0498	0.0123	0.0244
*Staphylococcus aureus*	0.0122	0.0249	0.0060	0.0122
*Enterococcus faecalis*	0.0122	0.0248	0.0028	0.0057

**Table 3:** The data of inhibition zone for opd and the nano-complex [Cu(opd)_2_(H_2_O) (μ-SCN)Ni(opd)(SCN)_3_] against investigated bacterial strains

	Inhibition zone, mm			
	opd	Nano-complex	Gentamycin	Amikacin
*Escherichia coli*	17	22	19.2	-
*Pseudomonas aeruginosa*	unavailable for the study	22	20.1	-
*Staphylococcus aureus*	17	28	-	18.8
*Enterococcus faecalis*	unavailable for the study	26	-	19.2

## Conclusion

In this study, a binuclear combination of Cu(II) and Ni(II) with formula [Cu(opd)_2_(H_2_O)(μ-SCN) Ni(opd)(SCN)_3_] has been synthesized and studied using UV-Vis and Fourier transform infrared spectroscopy. The solvothermal method was used to create the nanoscale of (a). X-ray powder diffraction (XRD) and scanning electron microscopy (SEM) methods were used to characterize the nanoparticles.

The inhibition and dilution methods were used to measure the minimum inhibitory concentration (MIC) and the lowest bactericidal concentration (MBC), which demonstrated that nano-complex (a) exhibits antibacterial properties. Two gram-positive bacteria, which included Staphylococcus aureus (*S. aureus ATCC* 25923) and *Enterococcus faecalis* (*Enter_faeca ATCC* 29212), together with two gram-negative bacteria, which included *Escherichia coli* (*E. coli* ATCC 25922) and Pseudomonas aeruginosa (*P. aeruginosa* ATCC 27853), were tested. The results showed that the nano-complex [Cu(opd)_2_(H_2_O)(μ-SCN) Ni(opd)(SCN)_3_] showed higher antimicrobial activity than ligand.

The Cu-Ni/SPGE system was used to conduct an electrochemical measurement of IRN. The Cu-Ni/SPGE outperformed the untreated SPGE in terms of IRN oxidation.
